# Celiac disease and the gut microbiota: bibliometric mapping of research hotspots and immune-mechanistic trends

**DOI:** 10.3389/fimmu.2026.1915319

**Published:** 2026-08-11

**Authors:** Shi Cheng, Shenglong Xue, Jinjin Xie, Yan Feng, Tian Shi, Feng Gao

**Affiliations:** 1 Department of Gastroenterology, People’s Hospital of Xinjiang Uygur Autonomous Region, Urumqi, China; 2 Xinjiang Clinical Research Center for Digestive Diseases, Urumqi, China; 3 College of Life Science and Technology, Xinjiang University, Urumqi, China

**Keywords:** bibliometric analysis, celiac disease, epithelial barrier, gluten-free diet, gut microbiota, probiotics

## Abstract

**Background:**

Celiac disease (CeD) is a chronic gluten-triggered immune-mediated enteropathy. Increasing evidence suggests that the gut microbiota may contribute to CeD pathophysiology. However, the global research landscape, hotspot evolution, and immune-mechanistic trends in CeD–gut microbiota research remain insufficiently mapped. This study aimed to systematically map the current research landscape and future hotspots in CeD–gut microbiota research.

**Methods:**

We conducted a bibliometric analysis with a mechanism-oriented narrative interpretation. Literature related to CeD and the gut microbiota was retrieved from the Web of Science Core Collection (WoSCC) and PubMed through April 25, 2026, without a lower publication-year restriction (the earliest eligible record was published in 2005). A total of 265 WoSCC records constituted the primary bibliometric dataset, while 320 PubMed records were analyzed separately for cross-database sensitivity comparison. CiteSpace, VOSviewer, and Bibliometrix were used to analyze publication trends, journals, authors, countries or regions, institutions, co-citation structures, keywords, and burst terms.

**Results:**

Annual publication output showed an overall upward trend in CeD–gut microbiota research, indicating sustained academic attention to this field. Core journal distribution suggested that this research area spans nutrition, microbiology, immunology, gastroenterology, and molecular biology. *Nutrients* was the most productive journal, with 37 publications. Sanz Y was the most productive author, with 18 publications, and Italy was the leading country, with 73 publications. Keyword co-occurrence and burst analyses further suggested that the field has gradually shifted from descriptive dysbiosis studies toward functional and immune-mechanistic investigations.

**Conclusion:**

This study systematically mapped the knowledge structure and evolutionary trends of global CeD–gut microbiota research. The findings indicate that research attention has expanded from early descriptive analyses of microbial composition toward functional and immune-related questions involving the gut microbiota–gluten peptide metabolism–epithelial barrier–mucosal immunity axis, which may provide a useful conceptual framework for interpreting the evolution of the literature. Future studies should further integrate prospective cohorts, multi-site sampling, multi-omics analyses, standardized dietary assessment, and immune phenotyping to investigate causal host–microbe relationships and evaluate the potential of microbiota-targeted nutritional and immunological strategies in CeD.

## 1 Introduction

Celiac disease (CeD) is a chronic immune-mediated enteropathy triggered by dietary gluten and characterized by small-intestinal mucosal inflammation, villous injury, and heterogeneous clinical manifestations (1, 2). In genetically susceptible individuals, particularly those carrying HLA-DQ2 and/or HLA-DQ8 haplotypes, gluten ingestion initiates immune responses central to CeD pathogenesis (3). Its pathogenesis involves multiple interconnected processes, including immunogenic gluten peptides, tissue transglutaminase-mediated peptide deamidation, HLA-restricted antigen presentation, and subsequent mucosal immune activation, ultimately resulting in small-intestinal inflammation and villous injury (4). Although lifelong adherence to a strict gluten-free diet (GFD) remains the cornerstone of CeD management, marked heterogeneity in disease onset, clinical phenotype, symptom persistence, and dietary responsiveness suggests that genetic susceptibility and gluten exposure, while central to disease development, are insufficient to fully explain CeD onset and progression (5, 6).

Increasing evidence suggests that the gut microbiota may act as an environmental and functional modifier within the gluten–epithelial barrier–mucosal immunity axis in CeD (7). As a key interface between dietary antigens and the host immune system, intestinal microbial communities may influence gluten peptide metabolism, epithelial barrier integrity, microbial metabolite production, and local immune regulation (8). Dysbiosis has been reported in fecal and duodenal samples from both pediatric and adult CeD cohorts, often reflected by alterations in microbial taxa with potential pro-inflammatory or immunomodulatory properties (9). Early-life cohort studies further suggest that HLA-associated genetic risk and familial susceptibility may shape gut microbial composition before clinical disease onset (10). Mechanistic studies have shown that selected duodenal bacteria can alter gluten degradation and peptide immunogenicity, while experimental models indicate that the gut microbiota can modulate gluten-induced immunopathology. Microbial metabolites, including short-chain fatty acids (SCFAs) and tryptophan-derived aryl hydrocarbon receptor (AhR) ligands, may contribute to epithelial homeostasis, inflammatory signaling, and T cell-associated immune balance, placing barrier integrity at an important interface between microbial alterations and mucosal immune activation (11). Nutritional and microbiota-targeted interventions, including probiotics, prebiotics, and GFD, further suggest that specific strains, nutritional substrates, and dietary patterns may modify the CeD-associated gut microbial environment through changes in microbial structure, barrier function, IgA- or Toll-like receptor-related mucosal responses, and metabolite production (12–14). However, findings remain heterogeneous across clinical populations, sampling sites, sequencing platforms, dietary exposures, and disease stages.

The literature on CeD and the gut microbiota has expanded rapidly in recent years. However, the knowledge structure of this field remains relatively fragmented, and studies differ in microbial alterations, sampling sites, sequencing methods, and disease status. Meanwhile, research attention has gradually extended from descriptive microbial profiling to functional and translational questions. Therefore, a bibliometric analysis with a mechanism-oriented narrative interpretation is needed to summarize the global research landscape, knowledge evolution, and immune-mechanistic trends in this field. Similar bibliometric approaches have been used to map research hotspots and trends in microbiome-related fields (15). Based on this rationale, the present study used CiteSpace, VOSviewer, and Bibliometrix to systematically analyze literature related to CeD and the gut microbiota in the Web of Science Core Collection (WoSCC) and PubMed databases up to April 25, 2026. This study aimed to construct a knowledge map and reveal global collaboration patterns, knowledge evolution, and emerging research frontiers.

## 2 Materials and methods

### 2.1 Study design and data sources

This study was designed as a bibliometric analysis with a mechanism-oriented narrative interpretation. WoSCC served as the primary bibliometric dataset, whereas PubMed was used for cross-database sensitivity analysis. Literature was retrieved through April 25, 2026, without a lower publication-year restriction.

### 2.2 Search strategy

To maximize retrieval, Medical Subject Headings (MeSH) terms from PubMed were used where applicable. The search strategy combined terms related to CeD and the gut microbiota and was adapted according to the search rules of WoSCC and PubMed. The main search terms included CeD, gut microbiota, and related terms. The complete search strategies are provided in the **Supplementary Material**.

### 2.3 Literature screening and inclusion criteria

This study included original research articles and review articles published in English. Records were included if they focused on CeD and involved gut microbiota, gut microbiome, dysbiosis, probiotics, prebiotics, microbial metabolites, or host–microbe interactions. Records were excluded if they were unrelated to CeD, referred to anatomical terms such as “celiac artery” or “celiac trunk” rather than celiac disease, lacked a gut microbiota-related component, or were editorials, letters, case reports, conference abstracts, non-English publications, or duplicates. Two researchers independently screened the records, and disagreements were resolved through discussion. After screening, 265 WoSCC records and 320 PubMed records were included. The detailed screening process is shown in **Supplementary Figure 1**.

### 2.4 Analysis methods

The WoSCC records were exported in plain text, tab-delimited, and BibTeX formats. CiteSpace (version 6.4.R1) was used to identify citation bursts in references, keywords, and countries, generate keyword clusters, and construct the dual-map overlay of journals. VOSviewer (version 1.6.18) was used to construct country collaboration, institutional collaboration, journal co-citation, author co-citation, and keyword co-occurrence networks. The Bibliometrix package in RStudio 4.2.3 was used to visualize source distribution, author productivity, and thematic structures. Microsoft Excel was used for data management and descriptive statistics. The PubMed dataset was used to compare publication trends and keyword patterns with those observed in WoSCC. In CiteSpace, nodes were selected using the g-index, and Pathfinder pruning was applied where applicable. Node size reflects frequency or citation counts, and links represent co-occurrence or co-citation relationships, with color indicating temporal distribution.

### 2.5 Mechanism-oriented thematic interpretation

To connect the bibliometric results with immunological interpretation, high-frequency keywords, burst keywords, and highly cited references were manually organized by the authors into five mechanism-oriented thematic domains. The thematic assignments were independently checked by two authors, and disagreements were resolved through discussion. These author-defined domains were used as a narrative framework to facilitate biological interpretation of the bibliometric findings and were distinct from the algorithm-derived clusters; the original keyword network structure was not altered. The five domains were: CeD-associated dysbiosis and microbial signatures; gluten-free diet and nutritional modulation of microbiota; probiotics, prebiotics, and microbiota-targeted intervention; epithelial barrier dysfunction and mucosal immune regulation; and microbial metabolites and immune signaling. These domains were used to interpret the evolution of CeD–gut microbiota research within the conceptual framework of the gut microbiota–gluten peptide metabolism–epithelial barrier–mucosal immunity axis. The study characteristics, evidence directness, and main limitations of representative references used in the thematic interpretation are summarized in **Supplementary Table 3**.

## 3 Results

### 3.1 Publication output and journal distribution

**Figure 1:**
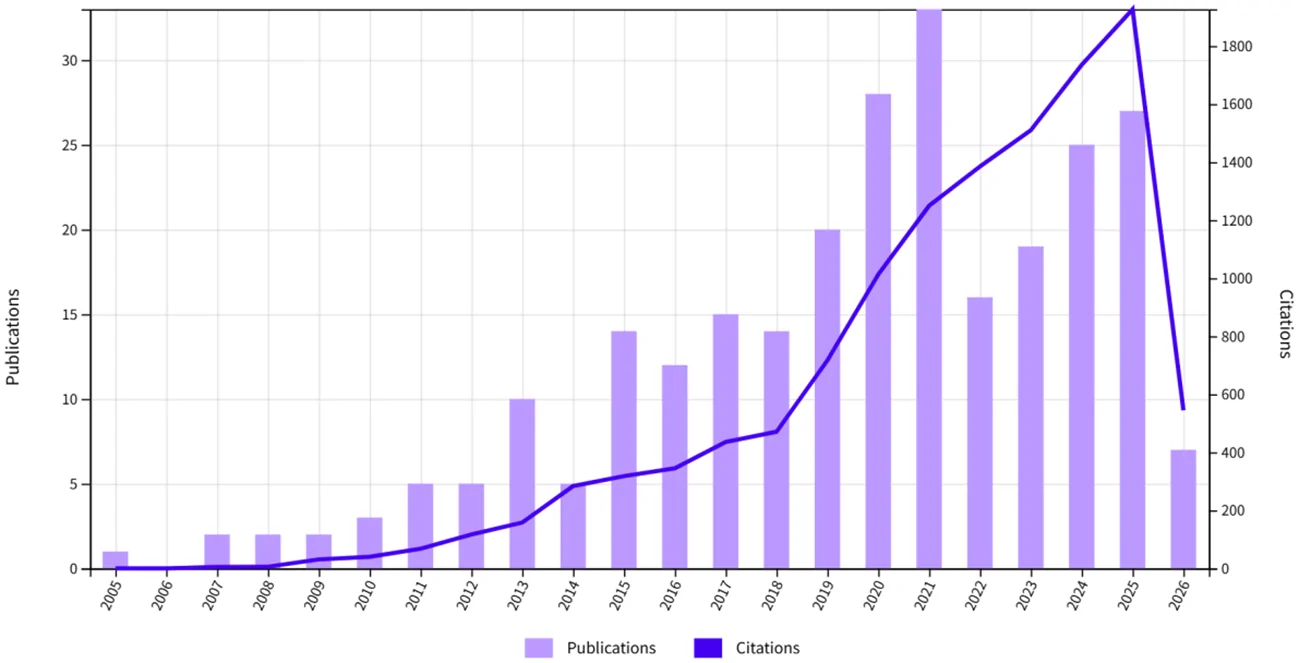
Annual publication and citation trends in the WoSCC dataset. Annual publication output and citation counts of studies on celiac disease and the gut microbiota in the Web of Science Core Collection dataset (n = 265). Records from 2026 were retrieved up to April 25, 2026 and therefore represent a partial publication year.

**Figure 2:**
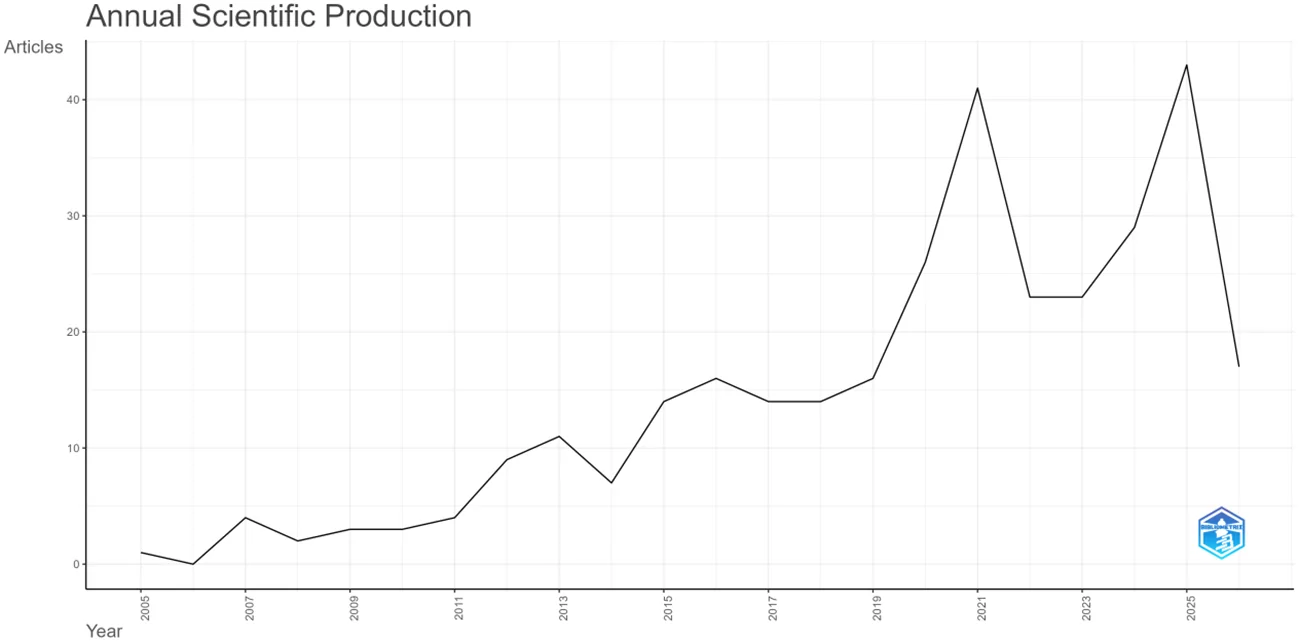
Annual publication trend in the PubMed dataset (sensitivity analysis). Annual scientific production of studies on celiac disease and the gut microbiota in the PubMed dataset (n = 320). Records from 2026 were retrieved up to April 25, 2026 and therefore represent a partial publication year.

A total of 265 WoSCC records were included in the primary analysis, and 320 PubMed records were included in the sensitivity analysis. The earliest eligible record identified by the topic-specific search was published in 2005. By measuring fecal short-chain fatty acids, this early study suggested intrinsic differences in gut microbial metabolic activity between children with CeD and healthy children, regardless of gluten-free diet treatment status (16). Overall, the annual publication output in the WoSCC dataset showed an upward trend, indicating increasing attention to CeD–gut microbiota research. The PubMed dataset showed a broadly similar publication trend to the WoSCC dataset (**Figure 1**, **2**).

**Figure 3:**
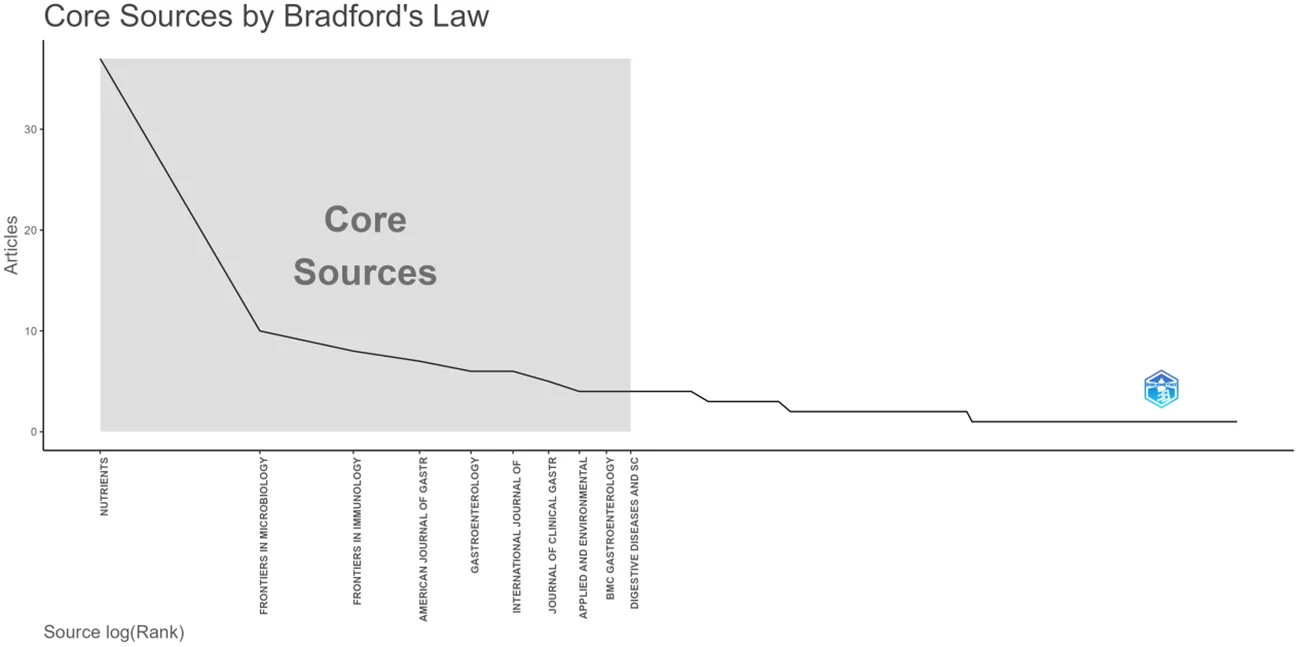
Core sources in the WoSCC dataset based on Bradford’s Law. Core journals contributing to celiac disease–gut microbiota research in the WoSCC dataset. The shaded area indicates the core source zone identified by Bradford’s Law.

**Figure 4:**
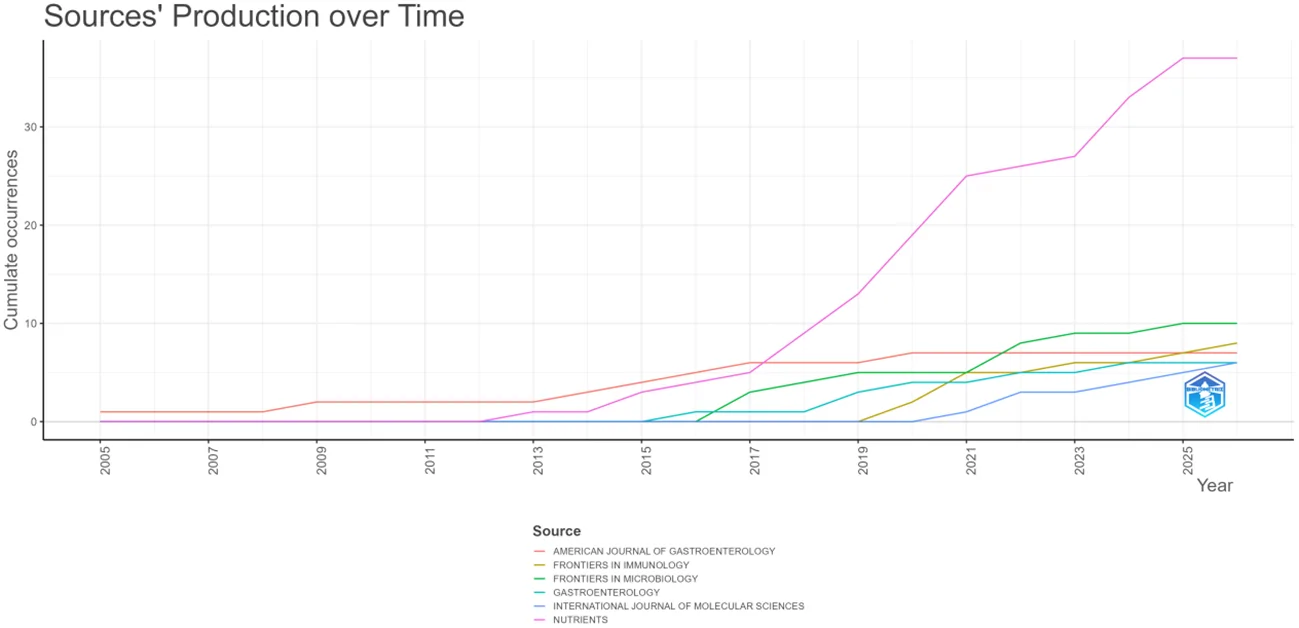
Temporal production of core journals in the WoSCC dataset. Cumulative publication output of the major core journals over time in the WoSCC dataset.

**Figure 5:**
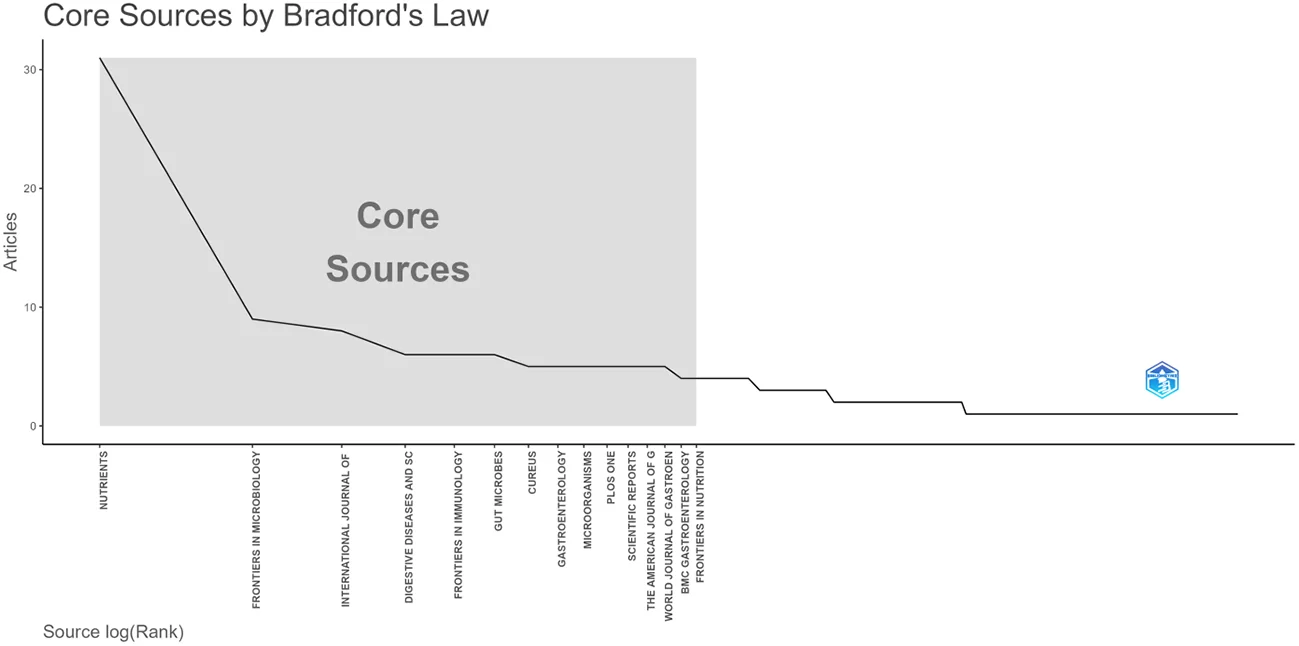
Core sources in the PubMed dataset (sensitivity analysis) based on Bradford’s Law. Core journals contributing to celiac disease–gut microbiota research in the PubMed dataset (sensitivity analysis). The shaded area indicates the core source zone identified by Bradford’s Law.

**Figure 6:**
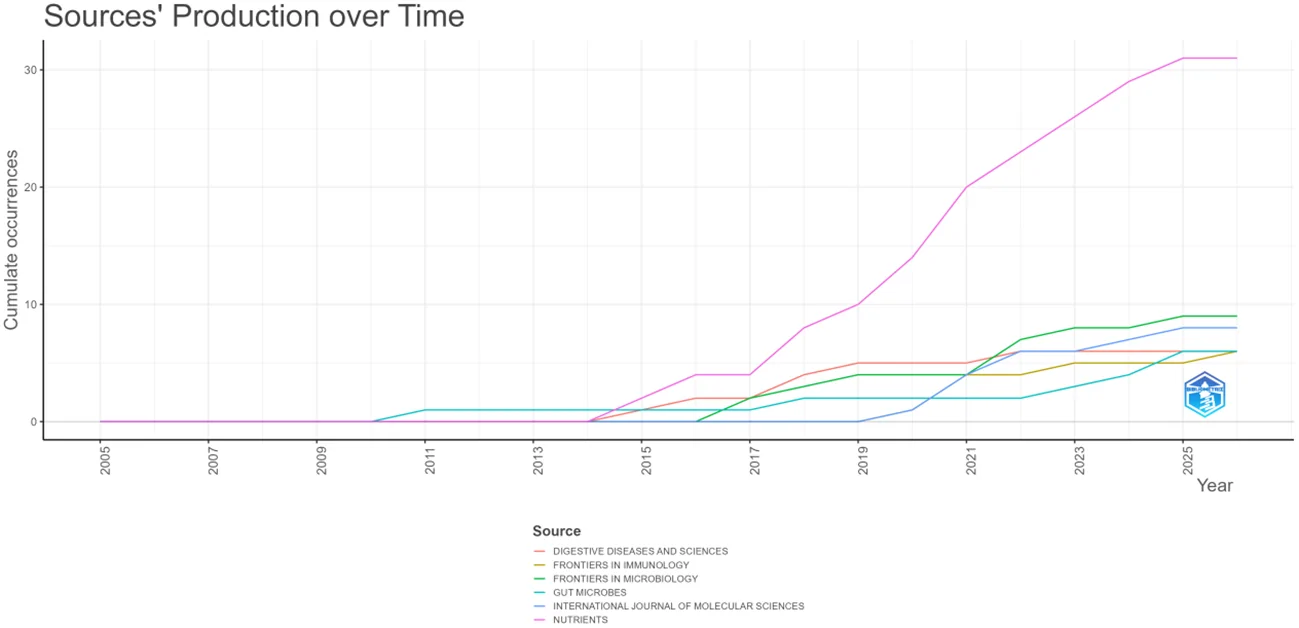
Temporal production of core journals in the PubMed dataset (sensitivity analysis). Cumulative publication output of the major core journals over time in the PubMed dataset (sensitivity analysis).

Journal distribution analysis showed that CeD–gut microbiota research was published across 141 journals. According to **Supplementary Table 1**, *Nutrients* ranked first, with 37 publications, accounting for 13.96% of the WoSCC dataset. This was followed by *Frontiers in Microbiology* with 10 publications, *Frontiers in Immunology* with 8 publications, *American Journal of Gastroenterology* with 7 publications, and *Gastroenterology* and *International Journal of Molecular Sciences* with 6 publications each (**Supplementary Table 1**). According to Bradford’s Law, specific journals constituted the core source zone, and their annual publication output increased over time, indicating sustained research activity in this field (**Figure 3**, **4**). The core journal distribution and temporal trends in the PubMed dataset were broadly comparable with those observed in the WoSCC dataset (**Figure 5**, **6**).

**Figure 7:**
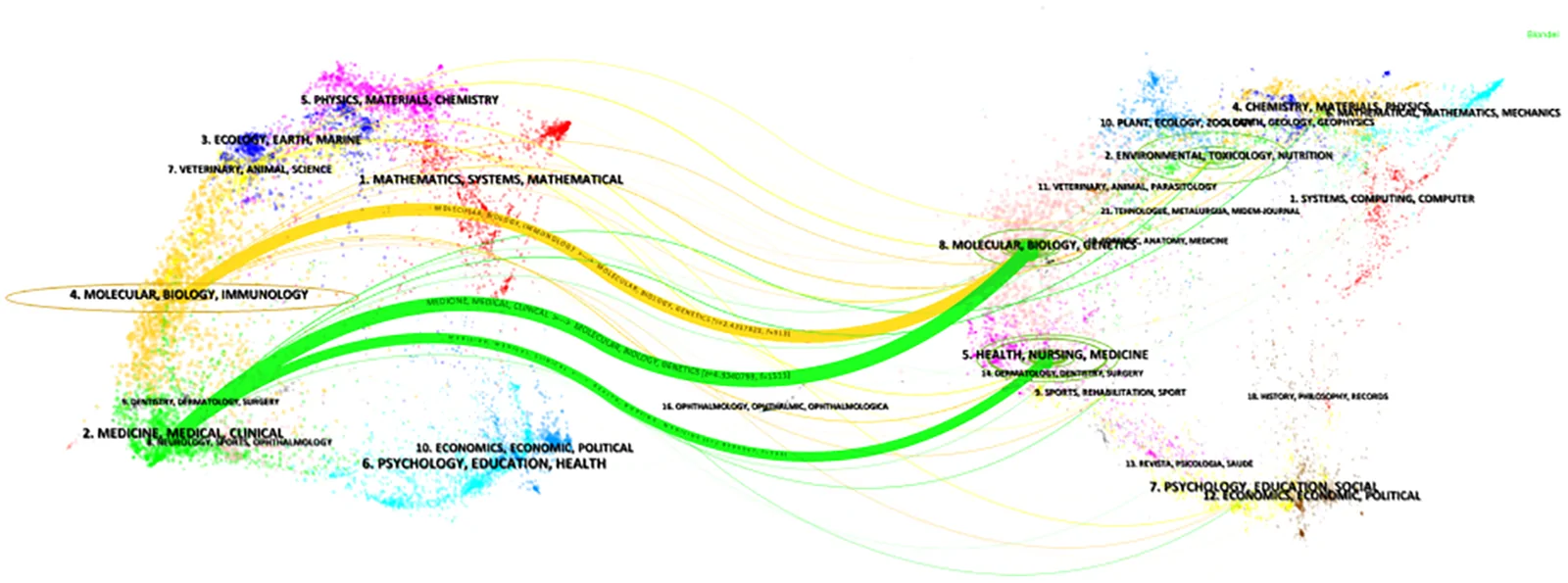
Dual-map overlay of journals in celiac disease and gut microbiota research. The left side represents the citing journals, while the right side represents the cited journals. Colored paths indicate citation relationships among different disciplines, revealing the interdisciplinary characteristics centered on molecular biology, genetics, and clinical medicine.

The predominance of *Nutrients* suggests that dietary modulation and nutritional intervention are among the core themes in CeD–gut microbiota research. Meanwhile, the presence of *Frontiers in Immunology* among the productive journals indicates that immune-mechanistic perspectives have become an important component of this field. In addition, the dual-map overlay analysis showed that citing journals were mainly concentrated in molecular biology, immunology, and clinical medicine, whereas cited journals were mainly distributed in molecular biology, genetics, and medicine (**Figure 7**). The major citation path from “Medicine, Medical, Clinical” to “Molecular Biology, Genetics” indicates that this field is rooted in clinical medicine while increasingly integrating molecular biology and genetics.

### 3.2 Author visualization analysis

**Figure 8:**
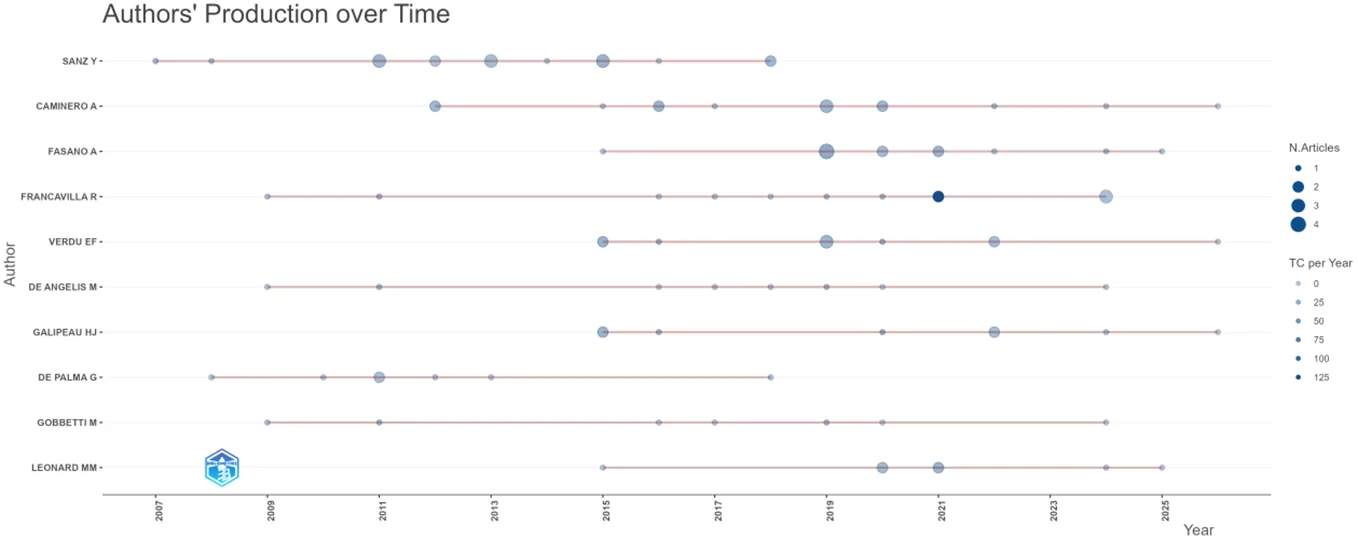
Temporal publication activity of the top ten authors in the WoSCC dataset. Bubble plot showing the publication activity of the top ten authors over time. Bubble size represents the number of publications, and color intensity indicates citation impact per year.

**Figure 9:**
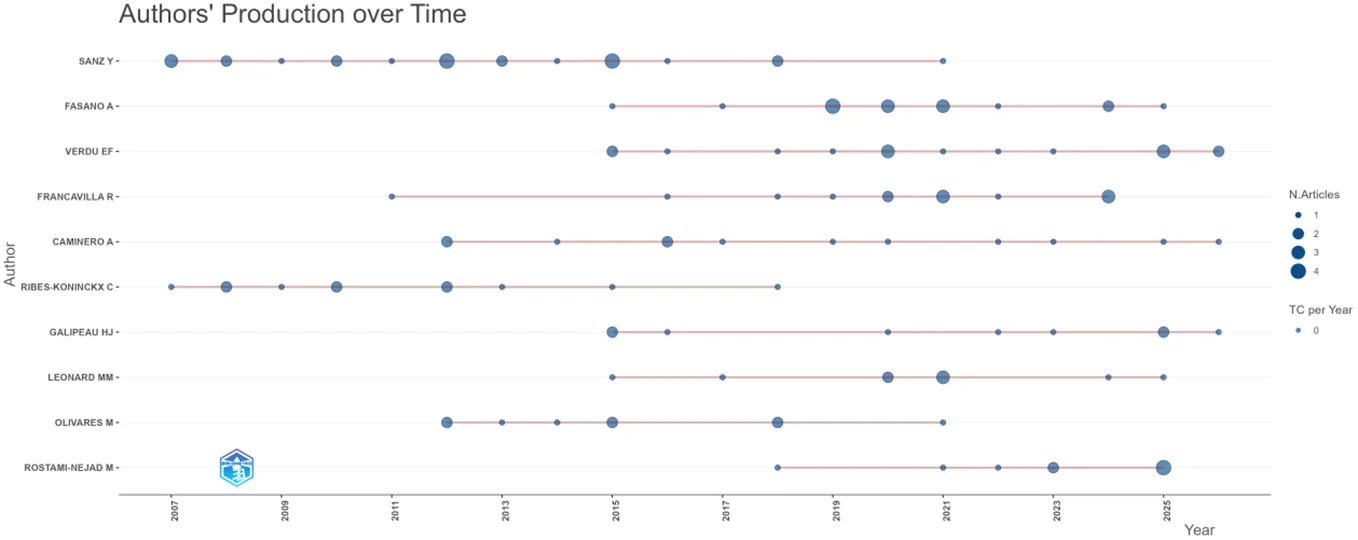
Temporal publication activity of the top ten authors in the PubMed dataset (sensitivity analysis). Bubble plot showing the publication activity of the top ten authors over time in the PubMed dataset (sensitivity analysis). Bubble size represents the number of publications.

Bibliometrix analysis showed that a total of 1,419 authors contributed to publications in the field of CeD and gut microbiota. The author co-citation network is shown in **Supplementary Figure 3**. The top ten authors by publication output in the WoSCC and PubMed datasets are shown in **Figure 8**, **9**, respectively. In the WoSCC dataset, Sanz Y was the most productive author, with 18 publications. These authors mainly contributed to studies involving pediatric CeD-associated dysbiosis, duodenal and fecal microbiota, gluten-free diet, probiotics or prebiotics, and host–microbe interactions (17–19). Given the involvement of nutrition, microbiology, immunology, and gastroenterology in CeD–gut microbiota research, the author network showed an interdisciplinary pattern rather than concentration within a single field.

### 3.3 Country or region and institutional cooperation analysis

**Figure 10:**
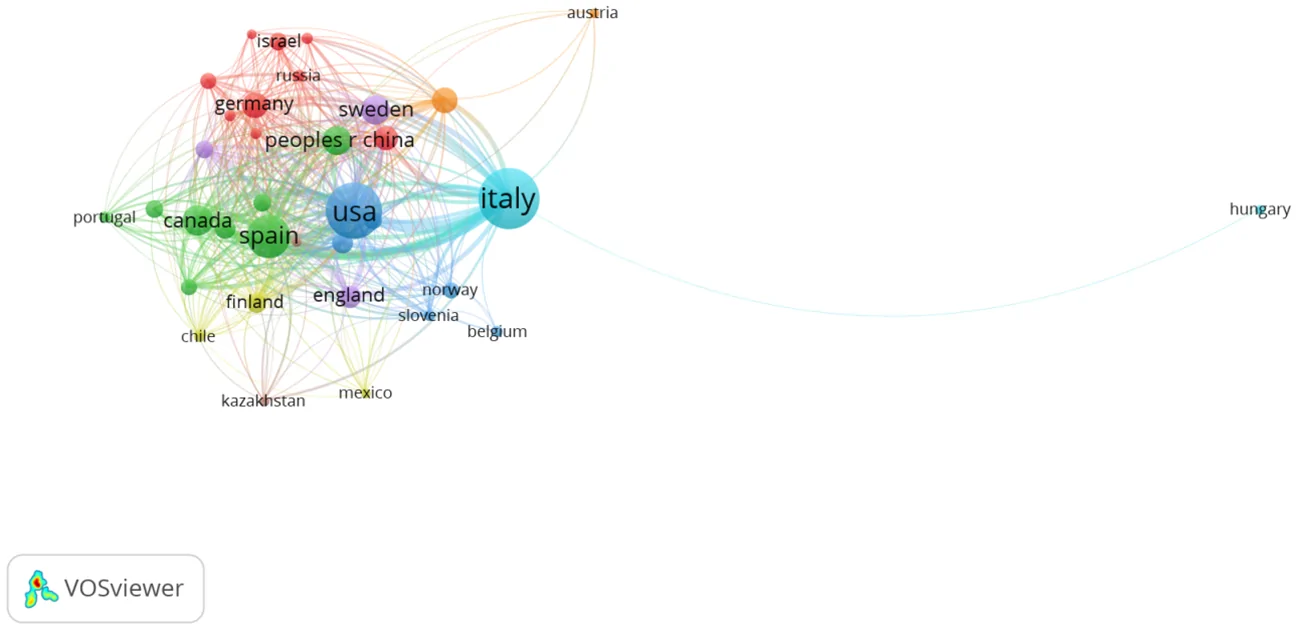
Network visualization of country-level collaboration in celiac disease–gut microbiota research generated using VOSviewer based on the WoSCC dataset. Countries with at least two publications were included. Full counting and association strength normalization were applied for network construction. Node size represents national publication output, links indicate collaboration relationships, and colors indicate collaboration clusters identified by the VOSviewer clustering algorithm.

**Figure 11:**
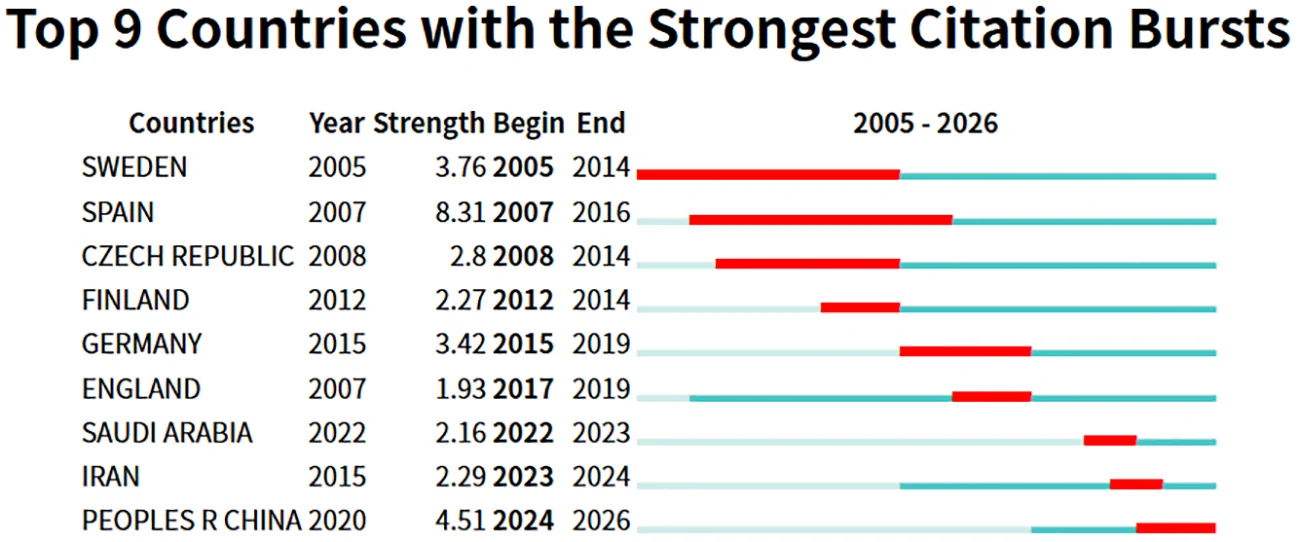
Burst countries in the WoSCC dataset. National citation burst analysis illustrates the temporal dynamics of major research contributors to celiac disease–gut microbiota research. Red bars denote citation burst periods, and those extending to the end of the timeline indicate countries or regions with continuing research activity.

**Figure 12:**
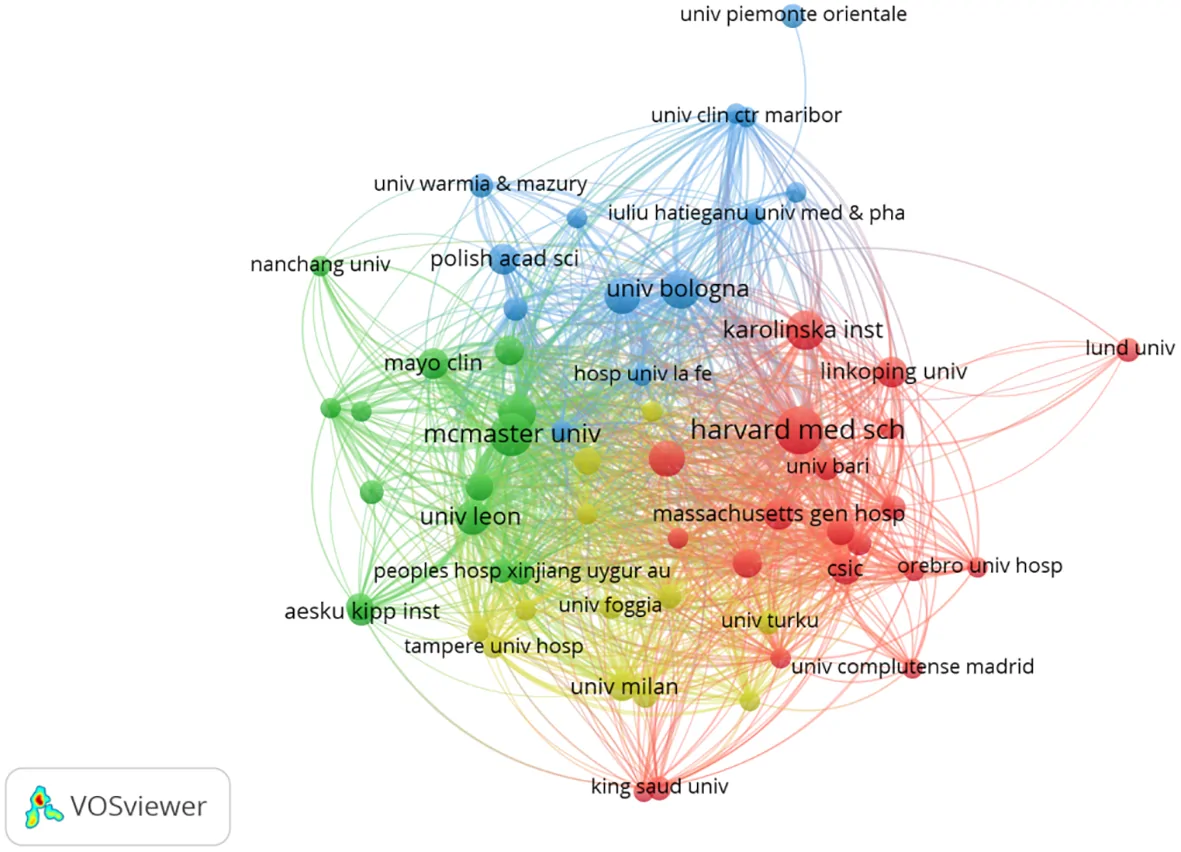
Network visualization of institutional collaboration in celiac disease–gut microbiota research generated using VOSviewer based on the WoSCC dataset. Institutions with at least three publications were included. Full counting and association strength normalization were applied for network construction. Node size represents institutional publication output, links indicate collaboration relationships, and colors indicate institutional collaboration clusters identified by the VOSviewer clustering algorithm.

Country or region collaboration analysis showed that CeD–gut microbiota research was distributed across multiple countries and regions (**Figure 10**). In the country collaboration network, node size represents publication output, whereas links represent collaboration strength. Italy was the leading country in terms of publication output, with 73 publications. Country burst analysis showed that several countries exhibited periods of strong research activity, suggesting that these countries may continue to be important contributors to this field (**Figure 11**). However, burst countries should be interpreted as indicators of temporal research activity rather than direct predictors of future dominance. Institutional collaboration analysis further revealed the major research institutions involved in CeD–gut microbiota studies (**Figure 12**).

### 3.4 Literature citation and co-citation analysis

Highly cited literature analysis revealed the major knowledge base of CeD–gut microbiota research. The top ten highly cited references are listed in **Supplementary Table 2**. The study designs, evidence directness, and main limitations of representative references are further summarized in **Supplementary Table 3**. Nadal et al. reported that patients with CeD showed an increase in opportunistic bacteria such as Bacteroides and Escherichia coli, together with a decrease in Lactobacillus and Bifidobacterium, suggesting that duodenal microbial imbalance may be associated with intestinal mucosal inflammation and disease phenotype (20). Schippa et al. further reported a distinctive microbial signature in pediatric CeD patients, supporting the presence of disease-associated microbial patterns in childhood CeD (21). Using combined microbiota and metabolomic analyses, Di Cagno et al. further showed that children with CeD receiving GFD treatment still had intestinal microbiota and metabolic abnormalities, including altered short-chain fatty acid metabolism and increased Gram-negative bacteria (22).

In adult CeD research, Wacklin et al. reported that patients with different clinical manifestations had distinct duodenal microbiota structures. Patients with gastrointestinal manifestations were mainly characterized by increased *Proteobacteria* and reduced microbial diversity, whereas the microbiota composition of patients with dermatitis herpetiformis was closer to that of healthy controls (23). Caminero et al. further demonstrated, using gnotobiotic mice, ex vivo barrier assays, and CeD patient T-cell assays, that selected duodenal bacterial strains generated distinct gluten degradation patterns and differed in the immunogenicity of the resulting peptides. Pseudomonas aeruginosa-modified peptides showed greater translocation across mouse intestinal tissue and activated gluten-specific T cells, whereas Lactobacillus strains further degraded these peptides and reduced their T-cell immunogenicity (24).

In addition, several highly cited reviews constituted an important theoretical foundation for this field. Verdu et al. summarized the potential role of gut microbiota in CeD development and progression, indicating that dysbiosis may already exist before disease onset and may persist after GFD treatment (25). Vancamelbeke et al. emphasized the key role of intestinal barrier integrity and tight junction abnormalities in CeD and other chronic intestinal inflammatory diseases (26). Cristofori et al. discussed the potential protective role of probiotics and gut microbiota in regulating CeD-related immune inflammation (27). Gasaly et al. systematically described how short-chain fatty acids, tryptophan metabolites, and bile acid metabolites may influence CeD-associated inflammation by regulating the intestinal barrier and mucosal immunity (28). Knezevic et al. proposed the concept of the “thyroid-gut axis”, suggesting that CeD and autoimmune thyroid diseases may share common mechanisms such as dysbiosis, increased intestinal permeability, and immune activation (29).

Burst reference analysis in the WoSCC dataset (**Supplementary Figure 4**) showed that Catassi et al. suggested that gut microbiota could influence gluten immunogenicity, epithelial barrier integrity, and immune activation processes. This finding suggests a shift in research paradigms from the traditional gluten–human leukocyte antigen-centered model toward a host–microbiome–immune interaction framework (30). Leonard et al. showed through birth cohort research that high-risk infants carrying HLA-DQ2/DQ8 genetic backgrounds already had alterations in gut microbiota composition and metabolic characteristics during early life, suggesting that host genetic factors may participate in early microbial colonization (31). Subsequently, longitudinal metagenomic research further showed that multiple pro-inflammatory microbial groups, metabolic pathways, and metabolites displayed dynamic abnormalities as early as 18 months before clinical CeD onset, whereas anti-inflammatory bacteria such as *Faecalibacterium prausnitzii* were significantly reduced (19). Finally, Zafeiropoulou et al. used 16S rRNA sequencing and metabolomic analysis to investigate alterations in intestinal microbiota in children with CeD at diagnosis and during GFD treatment, further supporting the importance of microbiota and metabolic changes in the disease process (32).

### 3.5 Keyword analysis and mechanism-oriented thematic domains

**Figure 13:**
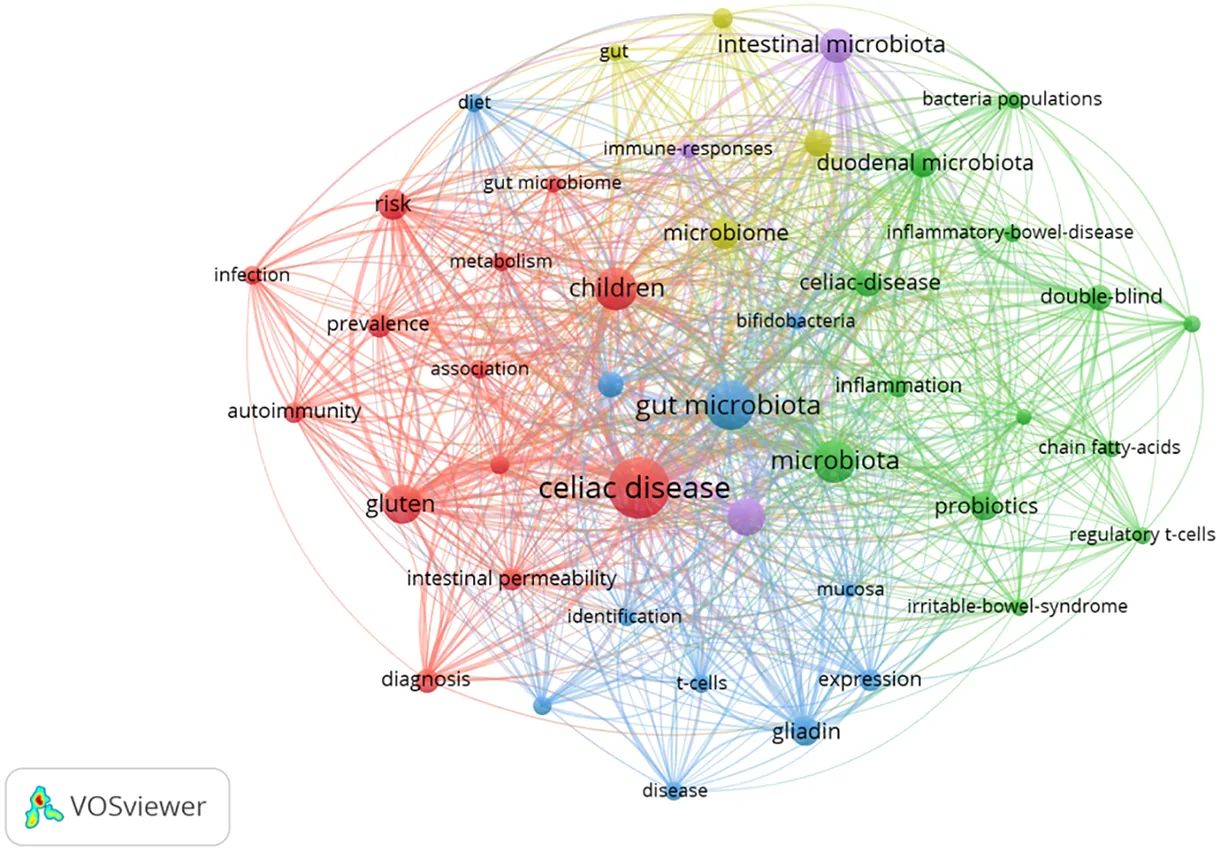
Network visualization of keyword co-occurrence in celiac disease–gut microbiota research generated using VOSviewer based on the WoSCC dataset. Keywords occurring at least 44 times were included. Full counting and association strength normalization were applied for network construction. Node size represents keyword occurrence frequency, links indicate co-occurrence relationships, and colors indicate keyword clusters identified by the VOSviewer clustering algorithm.

**Figure 14:**
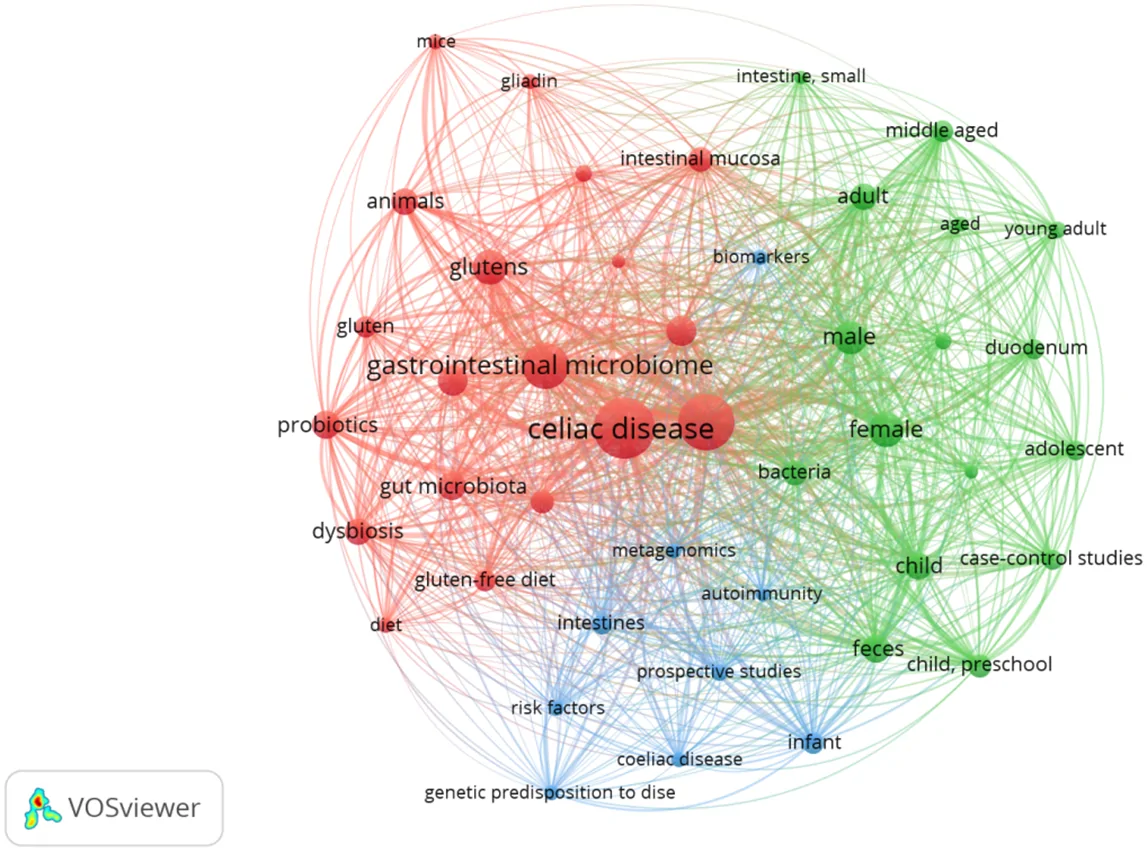
Network visualization of keyword co-occurrence in the PubMed sensitivity dataset generated using VOSviewer. Keywords occurring at least 44 times were included. Full counting and association strength normalization were applied for network construction. Node size represents keyword occurrence frequency, links indicate co-occurrence relationships, and colors indicate keyword clusters identified by the VOSviewer clustering algorithm.

**Figure 15:**
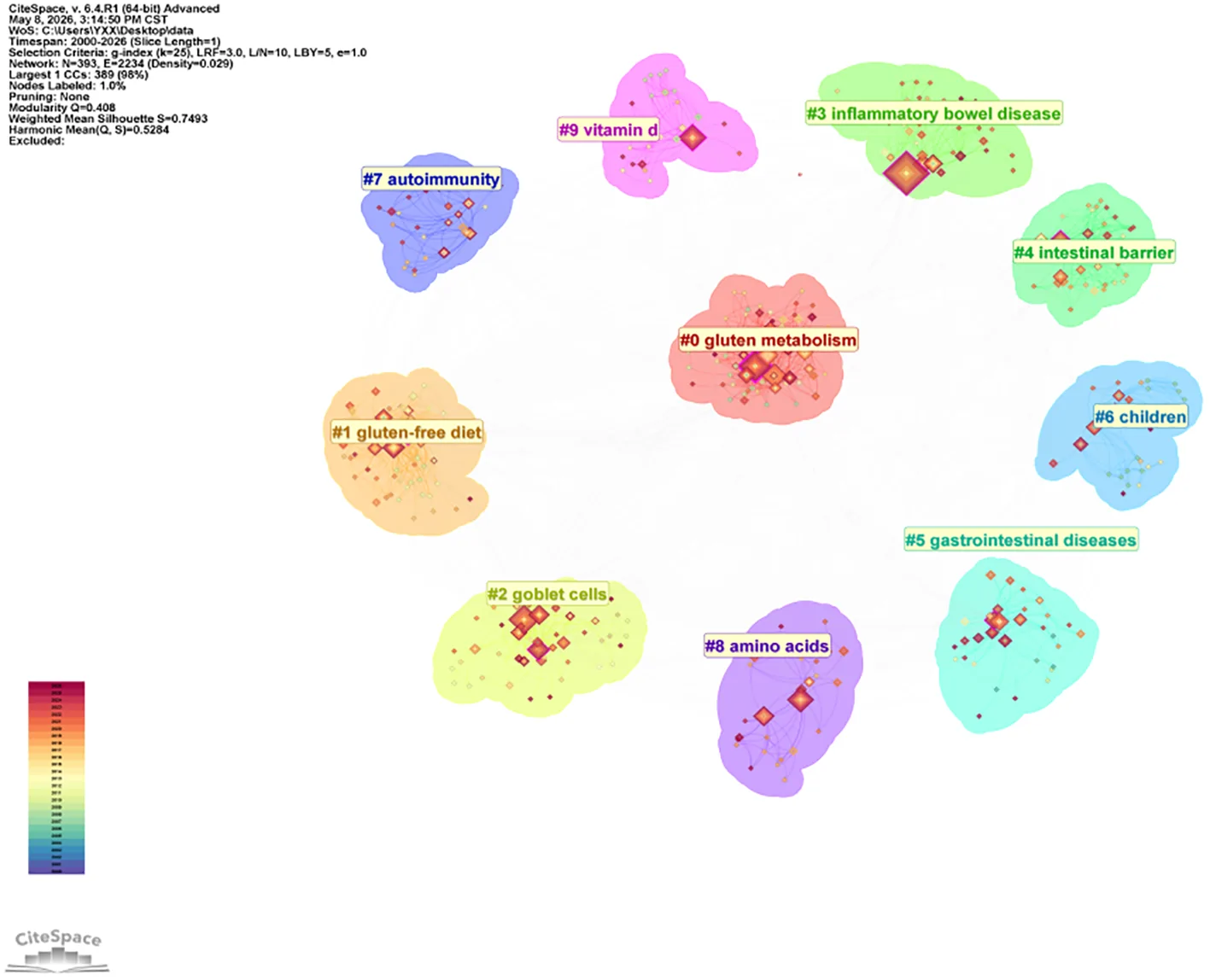
Keyword clustering network of the WoSCC dataset. Keyword clustering analysis was performed using CiteSpace based on the WoSCC dataset from 2005 to 2026 with one-year time slices. Keyword nodes were selected using the g-index method (k = 25), and cluster labels were generated using the log-likelihood ratio (LLR) algorithm. No pruning was applied. The modularity Q value was 0.408, and the weighted mean silhouette value was 0.7493. Node size indicates keyword frequency, links represent keyword co-occurrence relationships, node colors correspond to the temporal color scale, and the colored cluster regions and labels represent distinct keyword clusters.

Keyword co-occurrence results and clustering showed that CeD–gut microbiota research was organized around several interrelated hotspots (**Figures 13**–**15**). To further enhance the mechanistic interpretation of keyword analysis, this study summarized high-frequency keywords, burst keywords, and biologically related concepts into five mechanism-oriented thematic domains while retaining the original keyword co-occurrence network structure.

The first domain, CeD-associated dysbiosis and microbial signatures, included keywords such as celiac disease, gut microbiota, dysbiosis, inflammatory bowel disease, gastrointestinal diseases, and children. This domain reflects the descriptive foundation of the field, focusing on microbial alterations in fecal and duodenal samples from pediatric and adult CeD cohorts, as well as site-specific differences in the intestinal microbial ecosystem.

The second domain focused on gluten-free diet and nutritional modulation of microbiota. This domain included keywords such as gluten-free diet, gluten metabolism, and gluten. This theme suggests that GFD is not only a clinical management strategy for CeD but also an important factor influencing the intestinal microbial ecosystem and nutritional immune environment. Some microbiota alterations in patients with CeD may be mainly driven by GFD and changes in dietary fiber intake rather than by the disease itself, prompting researchers to distinguish between “disease-specific microbiota alterations” and “diet-related microbiota changes”.

The third domain focused on probiotics and microbiota-targeted intervention. Representative keywords included probiotics, diet, and vitamin D. This domain reflects researchers’ interest in microbiota-targeted adjunctive intervention strategies beyond GFD. Related studies found that some *Lactobacillus* and *Bifidobacterium* strains may reduce gluten peptide immunogenicity, regulate inflammatory responses, and improve intestinal mucosal homeostasis.

The fourth domain focused on epithelial barrier dysfunction and mucosal immune regulation. This domain included keywords such as intestinal barrier, goblet cells, and autoimmunity. This theme suggests that barrier function and mucosal immune regulation are important links connecting gut microbial ecological changes with the pathogenesis of CeD.

The fifth domain highlighted microbial metabolites and immune signaling. Representative keywords included short-chain fatty acids and amino acids. This theme suggests that the field is gradually shifting from purely taxonomic microbiota description toward functional interpretation based on microbial metabolites and immune signaling pathways.

Burst keyword analysis further showed that recent research attention has shifted toward functional and translational topics related to probiotics, supplementation, gluten-free diet, and other interventions (**Supplementary Figure 5**). Keyword co-occurrence and burst analyses suggest that recent CeD–gut microbiota research increasingly emphasizes functional and immune-related questions within the gut microbiota–gluten metabolism–epithelial barrier–mucosal immunity framework, together with growing research interest in early disease prediction and precision microbiota-targeted intervention. The biological implications of these themes are discussed further in Section 4.2.

## 4 Discussion

### 4.1 General information

This study systematically mapped the global research landscape of CeD–gut microbiota research based on WoSCC and PubMed. A total of 265 WoSCC records were included in the primary analysis, with 320 PubMed records used for sensitivity analysis. The two datasets showed broadly similar publication patterns. The annual publication trend showed an overall increase in research output, suggesting sustained attention to the potential role of gut microbiota in CeD development and progression.

From the perspective of journal distribution, CeD–gut microbiota research spans multiple disciplines, including nutrition, microbiology, immunology, gastroenterology, and molecular biology. This suggests that dietary modulation, microbiome biology, gastrointestinal disease mechanisms, and immune regulation jointly constitute the main disciplinary foundation of this field. In particular, both *Nutrients* and *Frontiers in Immunology* ranked among the productive journals, indicating that nutritional immunology and immune-mechanistic interpretation have become important directions in CeD–gut microbiota research.

Country and institutional maps showed broad international participation in CeD–gut microbiota research, although the geographic distribution of publication output did not appear to closely parallel reported CeD prevalence or diagnostic recognition. This pattern may reflect the different determinants of epidemiological estimates and scientific productivity: reported prevalence varies across regions and according to diagnostic definitions and case-ascertainment strategies (30, 33), with population-based screening revealing previously undiagnosed cases (34), whereas publication output is influenced by research capacity, funding, specialized research centers, international collaboration, and database coverage. Because microbiota findings may also vary with geography, dietary patterns, ethnicity, sample type, sequencing platform, and clinical phenotype (35), broader cross-regional, multicenter, and interdisciplinary collaborations are needed to improve reproducibility and generalizability.

Citation analysis showed that the CeD–gut microbiota literature has increasingly moved beyond the descriptive question of whether microbial composition differs in CeD toward functional and immune-related questions concerning microbial gluten peptide metabolism, epithelial barrier integrity, metabolite signaling, and mucosal immune regulation (20–29).

Keyword co-occurrence and burst analyses further supported this evolution in research emphasis. Taken together, these bibliometric patterns suggest that the gut microbiota–gluten peptide metabolism–epithelial barrier–mucosal immunity axis provides a useful framework for organizing the evolving research themes in this field.

### 4.2 Research hotspots

#### 4.2.1 CeD-associated dysbiosis and microbial signatures

CeD-associated dysbiosis and microbial signatures constitute the descriptive foundation of this field. Keyword co-occurrence networks and highly cited literature both suggest that microbial alterations in fecal and duodenal samples from pediatric and adult CeD cohorts have long been important research directions. Several influential studies have focused on duodenal microbiota imbalance and distinctive microbial signatures in pediatric patients with CeD, as well as the relationship between duodenal microbiota composition and clinical manifestations in adult patients with CeD. These findings support the view that gut microbiota may be associated with disease heterogeneity and host–microbe immune interactions.

Salamon et al. reported that newly diagnosed children with CeD and their asymptomatic siblings showed lower bacterial diversity than healthy children, while fungal communities differed more clearly between patients and siblings. Their study further identified differential bacterial and fungal signatures, suggesting that microbiota profiles in children with CeD and their siblings may serve as potential indicators of disease susceptibility (36). Wulczynski et al. found that fiber-degrading bacteria such as members of the *Prevotellaceae* family were reduced in the duodenum of patients with CeD, accompanied by impaired microbial glycolytic function and reduced short-chain fatty acid production. This defect was not fully restored even after long-term GFD, suggesting persistent dysfunction of small-intestinal microbial fiber metabolism in CeD (37).

However, dysbiosis should not be interpreted merely as a list of increased or decreased microbial taxa. Factors such as age, disease activity, dietary exposure, GFD adherence, sampling site, sequencing platform, and analytical pipeline may all influence microbiome results. Sampling site warrants particular attention because fecal microbial profiles may not fully reflect duodenal mucosa-associated communities at the primary site of CeD injury (25). Therefore, future studies should combine site-specific sampling with functional analyses to determine whether disease-associated microbial signatures are related to gluten peptide metabolism, epithelial barrier function, and mucosal immune regulation.

#### 4.2.2 GFD and nutritional modulation of microbiota

Gluten-free diet is not only the core strategy for the clinical management of CeD but also an important hotspot in CeD–gut microbiota research. GFD can reduce exposure to immunogenic gluten peptides and may also reshape the intestinal microbial ecosystem by altering dietary fiber intake, carbohydrate sources, micronutrient composition, and microbial substrate supply (38). Therefore, GFD should not merely be considered an antigen-removal strategy, but should also be understood as a nutritional intervention that may affect the microbiota–immune axis. Current studies suggest that GFD can alter gut microbiota composition, but it may not restore a healthy microbial state in all patients. This heterogeneity may be related to diet quality, GFD duration, disease stage, age, baseline microbiome composition, and dietary adherence. Future studies should combine standardized dietary assessment, microbiota sequencing, and immunological endpoints to further clarify how GFD affects intestinal microbial ecology and mucosal immune regulation (39, 40).

#### 4.2.3 Probiotics and microbiota-targeted intervention

Probiotics and prebiotics represent another important hotspot in this field, as supported by keyword co-occurrence networks, burst keywords, and highly cited literature. Highly cited studies on the anti-inflammatory and immunomodulatory effects of probiotics have been widely cited, indicating that microbiota-targeted intervention has become a potential adjunctive therapeutic strategy for CeD. Probiotics may influence gut microbial balance, epithelial barrier function, IgA- or Toll-like receptor-related mucosal immune responses, and microbial metabolite production (41). Some studies have suggested that *Lactiplantibacillus plantarum* and *Bifidobacterium* may reduce gluten-related immune activation by degrading immunogenic gluten peptides, improving epithelial barrier integrity, modulating inflammatory cytokines, and reshaping gut microbial composition (42, 43). These findings support the potential of probiotic strains as adjunctive strategies beyond GFD. However, current evidence remains heterogeneous and strain-specific, and further clinical validation is required. Existing intervention studies differ in strain selection, dosage, intervention duration, patient age, disease status, GFD adherence, and outcome measures (44). In addition, many studies mainly focus on microbiota composition changes but lack immunological or metabolomic endpoints. Future clinical trials should include strain-level identification, standardized dietary assessment, and mechanistically meaningful outcomes, such as epithelial permeability markers, IgA responses, T cell-related immune signatures, and microbial metabolite profiles. Such designs may help clarify whether probiotics or prebiotics can serve as effective adjunctive interventions beyond GFD.

#### 4.2.4 Epithelial barrier dysfunction and mucosal immune regulation

Highly cited literature and keyword analysis both indicate that epithelial barrier dysfunction is one of the important immune-mechanistic hotspots in CeD–gut microbiota research. In CeD, epithelial barrier dysfunction may promote the translocation of immunogenic gluten peptides and microbial products, thereby amplifying HLA-restricted antigen presentation and mucosal immune activation (45). Gut microbiota may participate in this process by affecting tight junction integrity, epithelial permeability, and local inflammatory status. For example, CeD-associated dysbiosis may impair epithelial barrier integrity and promote mucosal inflammation through mechanisms involving antimicrobial peptides and local immune signaling (46). Meanwhile, mucosal immune activation may also reshape the intestinal ecological niche and further alter microbiota composition (47). Therefore, the relationship among dysbiosis, barrier dysfunction, and immune activation is unlikely to be a simple linear relationship, but may instead constitute a dynamic interaction network.

#### 4.2.5 Microbial metabolites and immune signaling

Microbial metabolites and immune signaling constitute a rapidly developing theme in CeD–gut microbiota research. Microbial metabolites may provide a mechanistic bridge between microbial ecology and immune regulation. Short-chain fatty acids, butyrate, tryptophan-derived AhR ligands, bile acids, and other microbial metabolites may influence epithelial homeostasis, inflammatory signaling, immune tolerance, and T cell-related immune balance (48). For example, SCFAs may alleviate gluten-induced intestinal immune inflammation by strengthening the intestinal epithelial barrier, promoting Treg-mediated anti-inflammatory responses, and inhibiting NF-κB and inflammatory cytokines (49). This hotspot suggests that the field is shifting from taxonomic microbiome profiling toward functional microbiome interpretation. Identifying altered microbial taxa remains important, but microbial metabolites may better reflect the biological consequences of dysbiosis. Future research should further integrate metagenomics, metabolomics, and immune phenotyping to clarify how microbial metabolic pathways influence gluten peptide processing, epithelial barrier function, and mucosal immune homeostasis. In studies of GFD, probiotics, prebiotics, or other microbiota-targeted interventions, metabolite profiles may also serve as important biomarkers for evaluating intervention responses and patient stratification.

### 4.3 Research trends: from descriptive dysbiosis to functional mechanism investigation

Keyword results suggest that CeD–gut microbiota research is undergoing a conceptual transition from descriptive dysbiosis analysis toward functional and immune-mechanistic research. Early studies mainly addressed whether microbiota composition differed between patients with CeD and healthy controls. In recent years, research focus has gradually shifted toward studies examining the possible roles of microbial communities in gluten peptide degradation, epithelial barrier integrity, microbial metabolite signaling, and mucosal immune regulation. This thematic evolution has been accompanied by substantial advances in methods used to characterize the gut microbiota, from targeted measurements of microbial metabolites, fluorescence *in situ* hybridization, and culture-dependent or PCR-based community-fingerprinting approaches in earlier studies (16, 20–23) to high-throughput 16S rRNA gene sequencing and functional assays (24), and more recently to shotgun metagenomic sequencing, metabolomics, and integrated multi-omics approaches (19, 31, 32). Because these methods differ in taxonomic and functional resolution, findings generated across different periods should be compared cautiously.

Building on these advances, the first important future trend is more comprehensive multi-omics integration. Although 16S rRNA sequencing helps describe microbiota composition, it provides limited functional resolution. Metagenomics, metabolomics, transcriptomics, and immune phenotyping can help clarify how microbial communities influence metabolic pathways and immune responses. Such integrative research may identify functional microbial signatures that are more biologically meaningful than taxonomic alterations alone (50).

The second trend is longitudinal cohorts and causal inference. Cross-sectional studies cannot determine whether dysbiosis occurs before CeD onset or reflects disease activity, dietary treatment, or inflammation-related ecological changes. Future studies should prioritize longitudinal research in individuals carrying HLA-DQ2 or HLA-DQ8 risk haplotypes, especially preclinical high-risk cohorts, to clarify whether microbiota changes precede clinical disease onset (51).

The third trend is precision nutrition and microbiota-targeted intervention. Future studies may integrate HLA risk, age, disease stage, GFD adherence, microbiota structure, metabolite characteristics, and immune phenotypes into research designs. Such stratification strategies may help evaluate whether patient subgroups differ in their responses to probiotics, prebiotics, postbiotics, or personalized dietary interventions, thereby informing future precision-nutrition and microbiota-targeted immunological approaches in CeD.

### 4.4 Strengths and limitations

This study has several strengths. First, the study used a dual-database strategy combining WoSCC and PubMed. WoSCC provides citation and co-citation information suitable for identifying the knowledge base, whereas PubMed provides complementary biomedical coverage and was used to examine the sensitivity of the principal descriptive findings to database selection. Second, this study combined conventional bibliometric visualization with mechanism-oriented thematic interpretation. Unlike general bibliometric studies that only report publication output, journals, authors, and keywords, this study further integrated keyword co-occurrence, burst keywords, and highly cited literature into five mechanism-oriented themes, thereby connecting quantitative mapping results with the immunopathological mechanisms of CeD. Third, this study proposed the gut microbiota–gluten peptide metabolism–epithelial barrier–mucosal immunity axis as a structured interpretive framework, which helps understand the evolution of CeD–gut microbiota research.

This study also has several limitations. First, although WoSCC and PubMed are commonly used databases, this study did not include Scopus, Embase, or non-English databases, which may have led to the omission of some relevant studies. Second, this study included only English-language publications, which may introduce language bias. Third, citation-based analysis usually favors earlier publications, whereas recent high-quality studies may not yet be fully represented in co-citation networks due to insufficient citation accumulation. Fourth, keyword co-occurrence and burst analyses depend on the terminology used by authors; mechanistically similar studies may use different expressions and therefore be underestimated in keyword networks, while the author-defined thematic organization also involves some interpretive subjectivity. Fifth, bibliometric analysis cannot establish causal relationships between the gut microbiota and CeD, intervention efficacy, or clinical utility. Sixth, some highly cited references are not CeD-specific studies but come from broader research on intestinal barriers, probiotics, bacterial metabolites, or autoimmune diseases. Therefore, manual validation and cautious interpretation are necessary when translating bibliometric signals into CeD-specific mechanistic explanations. Seventh, the bibliometric analyses did not stratify the literature by sample source; studies based on fecal samples and those based on duodenal mucosal biopsies were therefore included in the same analyses. Because these samples represent distinct intestinal microbial niches, this may have obscured sample-site-specific research patterns and limited the biological comparability of the underlying evidence.

## 5 Conclusion

Using data from WoSCC and PubMed, this study conducted a bibliometric analysis with a mechanism-oriented narrative interpretation of global CeD–gut microbiota research. Based on 265 WoSCC records and a sensitivity analysis of 320 PubMed records, this study systematically identified publication trends, core journals, author contributions, country and institutional collaborations, intellectual bases, keyword hotspots, and emerging research frontiers in this field.

Current CeD–gut microbiota research increasingly emphasizes microbial function, nutritional modulation, epithelial barrier integrity, microbial metabolites, and mucosal immune regulation in addition to descriptive analyses of microbial composition. The gut microbiota–gluten peptide metabolism–epithelial barrier–mucosal immunity axis may serve as a useful conceptual framework for organizing this evolving literature.

Future studies should further strengthen prospective longitudinal cohorts, multicenter collaborations, multi-site sampling, multi-omics integration, standardized dietary assessment, and immune phenotyping to determine whether and how the gut microbiota contributes to CeD onset, progression, and treatment response. Patient stratification based on microbial, metabolic, and immune features may help evaluate precision-nutrition and microbiota-targeted immunological strategies as potential complements to GFD.

## Data Availability

The original contributions presented in the study are included in the article/**Supplementary Material**. Further inquiries can be directed to the corresponding author.
