# Supplementary material for: Celiac disease and the gut microbiota: bibliometric mapping of research hotspots and immune-mechanistic trends
Source: Front Immunol. 2026 Aug 11;17:1915319. doi: 10.3389/fimmu.2026.1915319 (PMC13503304; doi:10.3389/fimmu.2026.1915319)
Supplement: Supplementary file 1 [file Table1.docx]

**Celiac disease and the gut microbiota: bibliometric mapping of research hotspots and immune-mechanistic trends**

**Appendix 1**

**Celiac Disease and the gut microbiome term**

( Disease, Celiac or Celiac Disease or Gluten Enteropathy or Enteropathies, Gluten or Enteropathy, Gluten or Gluten Enteropathies or Gluten-Sensitive Enteropathy or Enteropathies, Gluten-Sensitive or Enteropathy, Gluten-Sensitive or Gluten-Sensitive Enteropathies or Gluten Sensitive Enteropathy or Celiac Sprue or Sprue, Celiac or Sprue, Nontropical or Nontropical Sprue or Sprue ) and (Gastrointestinal Microbiome OR Gastrointestinal Microbiomes OR Microbiome, Gastrointestinal OR Gut Microbiome OR Gut Microbiomes OR Microbiome, Gut OR Gut Microflora OR Microflora, Gut OR Gut Microbiota OR Gut Microbiotas OR Microbiota, Gut OR Gastrointestinal Flora OR Flora, Gastrointestinal OR Gut Flora OR Flora, Gut OR Gastrointestinal Microbiota OR Gastrointestinal Microbiotas OR Microbiota, Gastrointestinal OR Gastrointestinal Microbial Community OR Gastrointestinal Microbial Communities OR Microbial Community, Gastrointestinal OR Gastrointestinal Microflora OR Microflora, Gastrointestinal OR Gastric Microbiome OR Gastric Microbiomes OR Microbiome, Gastric OR Intestinal Microbiome OR Intestinal Microbiomes OR Microbiome, Intestinal OR Intestinal Microbiota OR Intestinal Microbiotas OR Microbiota, Intestinal OR Intestinal Microflora OR Microflora, Intestinal OR Intestinal Flora OR Flora, Intestinal OR Enteric Bacteria OR Bacteria, Enteric)
