# Supplementary material for: Celiac disease and the gut microbiota: bibliometric mapping of research hotspots and immune-mechanistic trends
Source: Front Immunol. 2026 Aug 11;17:1915319. doi: 10.3389/fimmu.2026.1915319 (PMC13503304; doi:10.3389/fimmu.2026.1915319)
Supplement: Supplementary file 2 [file Table2.docx]

**Celiac disease and the gut microbiota: bibliometric mapping of research hotspots and immune-mechanistic trends**

# **Supplementary Material**

Supplementary Figure 1. Literature retrieval and screening flowchart.

Supplementary Figure 2. Journal co-citation network in the WoSCC dataset.

Supplementary Figure 3. Author co-citation network in the WoSCC dataset.

Supplementary Figure 4. Burst references in the WoSCC dataset.

Supplementary Figure 5. Burst keywords in the WoSCC dataset.

Supplementary Table 1. Top ten most productive journals in the WoSCC dataset.

Supplementary Table 2. Top ten highly cited references in the WoSCC dataset.

Supplementary Table 3. Characteristics, evidence directness, and limitations of representative studies used in the mechanism-oriented thematic interpretation.

**Supplementary Figure 1.** Literature retrieval and screening flowchart. Flowchart summarizing the identification, screening, eligibility assessment, and final inclusion of records from the WoSCC and PubMed databases.

**Supplementary Figure 2.** Journal co-citation network in the WoSCC dataset. Network visualization of journal co-citation analysis generated using VOSviewer based on the WoSCC dataset. Journals cited at least twice were included. Full counting was used, and association strength normalization was applied for network mapping and clustering. Node size represents journal citation frequency, links indicate co-citation relationships, link thickness reflects co-citation strength, and colors indicate journal co-citation clusters identified by the VOSviewer clustering algorithm.

**Supplementary Figure 3.** Author co-citation network in the WoSCC dataset. Network visualization of author co-citation analysis generated using VOSviewer based on the WoSCC dataset. Authors cited at least three times were included. Full counting was used, and association strength normalization was applied for network mapping and clustering. Node size represents author citation frequency, links indicate co-citation relationships, link thickness reflects co-citation strength, and colors indicate author co-citation clusters identified by the VOSviewer clustering algorithm.

**Supplementary Figure 4.** Burst references in the WoSCC dataset. Burst reference analysis illustrates the temporal evolution of highly influential references in celiac disease–gut microbiota research. Red segments indicate periods during which references showed citation-burst activity; red segments extending to the end of the timeline indicate that the citation burst was still ongoing at the April 25, 2026 search cutoff.

**Supplementary Figure 5.** Burst keywords in the WoSCC dataset. Burst keyword analysis shows the temporal evolution of research frontiers in celiac disease–gut microbiota research. Red segments indicate periods during which keywords showed burst activity; red segments extending to the end of the timeline indicate that the keyword burst was still ongoing at the April 25, 2026 search cutoff.

**Supplementary Table 1.** Top ten most productive journals in the WoSCC dataset. The table lists the top ten journals ranked by publication output, including journal name, number of publications, percentage of the total WoSCC dataset, 2024 impact factor, and JCR quartile.

| **Ranking** | **Journal** | **Output** | **% of 265** | **IF 2024** | **JCR 2024** |
| --- | --- | --- | --- | --- | --- |
| 1 | NUTRIENTS | 37 | 13.96 | 5.0 | Q1 |
| 2 | FRONTIERS IN MICROBIOLOGY | 10 | 3.77 | 4.5 | Q1 |
| 3 | FRONTIERS IN IMMUNOLOGY | 8 | 3.02 | 5.9 | Q1 |
| 4 | AMERICAN JOURNAL OF GASTROENTEROLOGY | 7 | 2.64 | 8.1 | Q1 |
| 5 | GASTROENTEROLOGY | 6 | 2.26 | 25.9 | Q1 |
| 6 | INTERNATIONAL JOURNAL OF MOLECULAR SCIENCES | 6 | 2.26 | 4.9 | Q1 |
| 7 | JOURNAL OF CLINICAL GASTROENTEROLOGY | 5 | 1.89 | 2.7 | Q2 |
| 8 | APPLIED AND ENVIRONMENTAL MICROBIOLOGY | 4 | 1.51 | 3.7 | Q2 |
| 9 | BMC GASTROENTEROLOGY | 4 | 1.51 | 2.6 | Q2 |
| 10 | DIGESTIVE DISEASES AND SCIENCES | 4 | 1.51 | 2.5 | Q2 |

**Supplementary Table 2.** Top ten highly cited references in the WoSCC dataset. The table lists the top ten cited references ranked by total citation counts, including title, first author, publication year, total citations, and citations per year.

| **Ranking** | **Title** | **First author** | **Year** | **Citations** | **Citations/Year** |
| --- | --- | --- | --- | --- | --- |
| 1 | The intestinal barrier: a fundamental role in health and disease | Vancamelbeke M | 2017 | 1033 | 103.3 |
| 2 | Anti-Inflammatory and Immunomodulatory Effects of Probiotics in Gut Inflammation: A Door to the Body | Cristofori F | 2021 | 672 | 112.0 |
| 3 | Impact of Bacterial Metabolites on Gut Barrier Function and Host Immunity: A Focus on Bacterial Metabolism and Its Relevance for Intestinal Inflammation | Gasaly N | 2021 | 435 | 72.5 |
| 4 | Imbalance in the composition of the duodenal microbiota of children with coeliac disease | Nadal I | 2007 | 318 | 15.9 |
| 5 | Thyroid-Gut-Axis: How Does the Microbiota Influence Thyroid Function? | Knezevic, J | 2020 | 266 | 38.0 |
| 6 | Duodenal and faecal microbiota of celiac children: molecular, phenotype and metabolome characterization | DiCagno, R | 2011 | 249 | 16.6 |
| 7 | A distinctive 'microbial signature' in celiac pediatric patients | Schippa S | 2010 | 191 | 11.2 |
| 8 | Novel players in coeliac disease pathogenesis: role of the gut microbiota | Verdu EF | 2015 | 184 | 15.3 |
| 9 | Duodenal Bacteria From Patients With Celiac Disease and Healthy Subjects Distinctly Affect Gluten Breakdown and Immunogenicity | Caminero A | 2016 | 182 | 16.6 |
| 10 | The Duodenal Microbiota Composition of Adult Celiac Disease Patients Is Associated with the Clinical Manifestation of the Disease | Wacklin P | 2013 | 155 | 11.1 |

**Supplementary Table 3.** Characteristics, evidence directness, and limitations of representative studies used in the mechanism-oriented thematic interpretation.

| **Reference** | **Thematic domain** | **Study design** | **Population / model** | **Sample size** | **Sample site** | **Analytical platform** | **Main findings** | **Directness to CeD** | **Main limitations** |
| --- | --- | --- | --- | --- | --- | --- | --- | --- | --- |
| **A. Primary empirical studies** | | | | | | | | | |
| 20. Nadal I, et al. (2007). *Imbalance in the composition of the duodenal microbiota of children with coeliac disease.* J Med Microbiol 56:1669-1674. | CeD-associated dysbiosis and microbial signatures | Cross-sectional comparative human observational study | Children with active CeD; symptom-free children with CeD on GFD for 1-2 years; non-CeD controls | n = 38: active CeD 20; GFD-treated CeD 10; controls 8 | Duodenal biopsy specimens | Group-specific fluorescent in situ hybridization (FISH) coupled with flow cytometry | Active CeD showed higher total and Gram-negative bacteria and higher proportions of *Bacteroides-Prevotella* and *Escherichia coli* than controls. The ratio of *Lactobacillus* + *Bifidobacterium* to *Bacteroides-Prevotella* + *E. coli* was reduced in both active and treated CeD; several deviations were attenuated in symptom-free GFD-treated patients. | Direct human CeD evidence; observational association | Small pediatric sample; cross-sectional groups rather than within-person follow-up; targeted probes covered selected bacterial groups only; no broad functional profiling; diet, inflammation, and disease effects could not be separated; no causal inference. |
| 21. Schippa S, et al. (2010). *A distinctive 'microbial signature' in celiac pediatric patients.* BMC Microbiol 10:175. | CeD-associated dysbiosis and microbial signatures; GFD-related modulation | Prospective longitudinal within-subject observational study with a control group | Children with CeD studied at diagnosis and after at least 9 months of GFD; non-CeD children undergoing endoscopy for functional dyspepsia | 30 unique participants; 50 biopsy observations: 20 active, 20 remission, 10 controls | Second-part duodenal biopsies | 16S rDNA PCR-temporal temperature gradient gel electrophoresis (TTGE); species-specific PCR; principal component, factorial discriminant, and partial least-squares discriminant analyses of fingerprint profiles | Dominant duodenal profiles differed between CeD and controls. Active CeD showed higher band-based diversity than remission and controls; *E. coli* and *Bacteroides vulgatus* were more prevalent in CeD. Profiles changed after GFD but usually remained distinct from controls. | Direct longitudinal human CeD evidence; observational association without causal inference | Small cohort; controls had functional dyspepsia; TTGE provides low taxonomic resolution and semi-quantitative band profiles; most patients lacked complete histologic normalization; high apparent classification accuracy was not externally validated; limited control of diet and other confounders. |
| 22. Di Cagno R, et al. (2011). *Duodenal and faecal microbiota of celiac children: molecular, phenotype and metabolome characterization.* BMC Microbiol 11:219. | Dysbiosis and microbial signatures; GFD/nutritional modulation; microbial metabolites | Cross-sectional multi-platform human observational study | Symptom-free children with treated CeD on GFD for at least 2 years; non-CeD children undergoing endoscopy for functional dyspepsia | n = 34: treated CeD 19; controls 15 | Duodenal biopsies, feces, and urine | Culture; 16S PCR-DGGE; group-specific PCR; strain identification and typing; SPME–GC–MS and ¹H NMR metabolomics | Treated CeD retained differences in duodenal and fecal microbial profiles and cultivable taxa. Feces showed lower *Lactobacillus*, *Enterococcus*, and *Bifidobacterium* and higher presumptive Gram-negative/opportunistic groups; volatile compounds, SCFAs, free amino acids, and urinary/fecal metabolomes also differed, suggesting incomplete normalization despite long-term GFD. | Direct human CeD evidence; exploratory cross-sectional association | Small sample; no untreated active-CeD group; controls had functional dyspepsia; long-term GFD was a major confounder; older low-resolution fingerprint and culture methods; many exploratory endpoints without external validation; cross-sectional design precluded temporal or causal inference. |
| 23. Wacklin P, et al. (2013). *The Duodenal Microbiota Composition of Adult Celiac Disease Patients Is Associated with the Clinical Manifestation of the Disease.* Inflamm Bowel Dis 19:934-941. | CeD-associated dysbiosis, microbial signatures, and clinical phenotype heterogeneity | Cross-sectional, phenotype-stratified human observational study | Untreated adults with newly diagnosed CeD (GI symptoms, dermatitis herpetiformis, anemia, screen-detected asymptomatic, or mixed phenotypes) and dyspepsia controls | n = 51: CeD 33 and controls 18; clone-sequencing subset n = 20, with the anemia subgroup excluded from sequence-based analysis because of low sequence yield | Distal duodenal biopsy specimens | Nested 16S rRNA PCR-DGGE; Shannon diversity and PCA; 16S rRNA clone-library sequencing and UniFrac in a subset | Duodenal community structure and diversity varied by clinical phenotype. GI-symptom CeD was enriched in Proteobacteria and showed lower diversity than dermatitis herpetiformis; dermatitis herpetiformis profiles were closer to controls. | Direct human CeD evidence; clinical-phenotype association | Small and uneven phenotype subgroups; cross-sectional design; dyspepsia rather than healthy controls; low-depth clone sequencing in a subset with failed amplifications/exclusions; older low-resolution methods; limited adjustment for diet, medications, and other confounders; no causal inference. |
| 24. Caminero A, et al. (2016). *Duodenal Bacteria From Patients With Celiac Disease and Healthy Subjects Distinctly Affect Gluten Breakdown and Immunogenicity.* Gastroenterology 151:670-683. | Microbial gluten metabolism; epithelial barrier and mucosal immune regulation; commensal/pathobiont functions | Translational mechanistic study combining in vitro bacterial assays, gnotobiotic mice, ex vivo barrier testing, and human T-cell assays | Duodenal bacterial isolates from CeD and non-CeD donors; germ-free C57BL/6 mice colonized with selected strains; HLA-DQ2.5+ biopsy-proven CeD participants in remission undergoing a 3-day gluten challenge | Human CeD n = 20 recruited (n = 10 in the key peptide ELISpot analysis); mice n = 13/group in the main colonization experiment; Ussing chamber n = 10/group | Human duodenal isolates and PBMCs; mouse small-intestinal contents and jejunum | Culture and gnotobiotic colonization; 16S sequencing; G12 ELISA; LC-MS/MS; IFN-gamma ELISpot; Ussing chambers; transposon mutant screening | *Pseudomonas aeruginosa* from CeD generated shorter gliadin peptides that retained immunogenicity, crossed mouse jejunal tissue more readily, and activated gluten-specific human T cells. *Lactobacillus* isolates from controls further degraded these peptides and reduced immunogenicity. | Direct CeD-relevant mechanistic evidence, but largely preclinical/ex vivo; human functional validation, not a clinical intervention | Reductionist selected-strain models did not reproduce complex human communities; selected cultivable isolates may not reflect in vivo abundance or function; cross-species extrapolation; small human functional subgroup; short gluten challenge and surrogate immune/barrier outcomes; no evidence of clinical efficacy or disease prevention. |

**Supplementary Table 3 (continued).** **Secondary evidence and contextual reviews.**

| **Reference** | **Thematic domain** | **Study design** | **Population / model** | **Sample size** | **Sample site** | **Analytical platform** | **Main findings** | **Directness to CeD** | **Main limitations** |
| --- | --- | --- | --- | --- | --- | --- | --- | --- | --- |
| **B. Secondary evidence and contextual reviews** | | | | | | | | | |
| 25. Verdu EF, et al. (2015). *Novel players in coeliac disease pathogenesis: role of the gut microbiota.* Nat Rev Gastroenterol Hepatol 12:497-506. | Overarching CeD microbiota-pathogenesis framework: dysbiosis, barrier, gluten metabolism, and immunity | CeD-focused narrative review | Human CeD observational cohorts, early-life risk studies, in vitro work, and animal/gnotobiotic models | Not applicable (secondary literature synthesis) | Fecal and duodenal/mucosal microbiota; experimental tissues and models | Narrative synthesis of culture-independent microbiota studies, clinical cohorts, and mechanistic models | Summarized evidence that microbial alterations may precede CeD, persist during GFD, and modulate gluten metabolism, barrier function, and innate/adaptive immunity. It emphasized the absence of a reproducible CeD microbial signature and the need for causal gnotobiotic and longitudinal studies. | CeD-specific secondary evidence; combines direct human associations with indirect mechanistic evidence | Narrative rather than systematic evidence synthesis; no pooled estimates or formal risk-of-bias assessment; underlying studies were heterogeneous in age, sampling site, method, GFD duration, and phenotype; causality and therapeutic implications remained unresolved. |
| 26. Vancamelbeke M, Vermeire S. (2017). *The intestinal barrier: a fundamental role in health and disease.* Expert Rev Gastroenterol Hepatol 11:821-834. | Epithelial barrier dysfunction and mucosal immune regulation | Narrative/expert review based on an extensive PubMed search | Barrier biology across IBD, CeD, nonalcoholic fatty liver disease, and other conditions; human, ex vivo, in vitro, and animal evidence | Not applicable (secondary literature synthesis) | Intestinal mucosa/biopsies, blood, urine, feces, cell models, and animal tissues | Narrative synthesis of barrier anatomy and permeability assays, including sugar tests, Ussing chambers, TEER, biomarkers, and imaging | Defined structural and immune components of the intestinal barrier and reviewed methods to assess permeability and barrier function. CeD was discussed as one disease linked to barrier defects, with particular emphasis on poor comparability across methods. | Indirect/contextual for CeD; not a CeD-specific review | No CeD-specific systematic search or quantitative synthesis; evidence spanned multiple diseases and methods; permeability and barrier-function measures were not interchangeable; assay validity and cross-study comparability were limited; therapeutic claims required larger trials. |
| 27. Cristofori F, et al. (2021). *Anti-Inflammatory and Immunomodulatory Effects of Probiotics in Gut Inflammation: A Door to the Body.* Front Immunol 12:578386. | Probiotics, prebiotics, and microbiota-targeted intervention | Broad narrative review | In vitro/ex vivo systems, animal models, and human studies across CeD, IBD, IBS, obesity, autism, Parkinson disease, and other inflammatory conditions | Not applicable (secondary literature synthesis) | Mixed intestinal tissues, cell cultures, stool, blood/PBMCs, and animal models | Narrative synthesis of probiotic strain, immune-signaling, barrier, postbiotic, and clinical evidence | Described strain-specific effects on TLR/NF-kappaB/MAPK signaling, cytokines, Treg/Th responses, IgA, and barrier function. It summarized small CeD studies suggesting changes in inflammatory markers, symptoms, or microbiota, while recognizing that GFD remains the only proven therapy. | Mixed: a CeD-specific subsection includes some human intervention evidence, but much mechanistic support is non-CeD or preclinical | Narrative selection; highly heterogeneous diseases, strains, doses, models, and outcomes; no formal risk-of-bias assessment or pooled CeD effect; mechanisms were often extrapolated from non-CeD models; diet, age, BMI, medications, and stress could confound probiotic effects; clinical efficacy was not established. |
| 28. Gasaly N, et al. (2021). *Impact of Bacterial Metabolites on Gut Barrier Function and Host Immunity: A Focus on Bacterial Metabolism and Its Relevance for Intestinal Inflammation.* Front Immunol 12:658354. | Microbial metabolites and immune signaling | Broad narrative mechanistic review | Human and experimental literature on intestinal inflammation, primarily IBD, with selected CeD evidence | Not applicable (secondary literature synthesis) | Intestinal epithelium, immune cells, stool/serum, and animal/cell models | Narrative synthesis of SCFAs, dietary fibers, tryptophan/AhR metabolites, and bile acid-FXR/TGR5 pathways | Outlined how SCFAs, indoles/AhR ligands, and secondary bile acids can regulate tight junctions, epithelial stress, Treg/IL-10/IL-22 pathways, and inflammatory signaling. Direct CeD data were limited and sometimes inconsistent, including SCFA findings. | Mostly indirect/contextual for CeD; mechanistic rationale largely derived from IBD, animal, and cell studies | Not CeD-specific; narrative rather than formal systematic synthesis; substantial reliance on preclinical and IBD evidence; metabolite effects were dose-, site-, and context-dependent; few direct human CeD studies; therapeutic applicability remained hypothetical. |
| 29. Knezevic J, et al. (2020). *Thyroid-Gut-Axis: How Does the Microbiota Influence Thyroid Function?* Nutrients 12:1769. | Epithelial barrier and mucosal immunity in the thyroid-gut axis; micronutrients and probiotics | Narrative literature review | Studies of autoimmune thyroid disease, thyroid cancer, bariatric surgery, CeD/non-celiac wheat sensitivity, animal models, and human cohorts | Not applicable (secondary literature synthesis) | Gut-thyroid axis; intestinal barrier, blood, stool, and animal models | Narrative synthesis of microbiota, intestinal permeability, micronutrient, and probiotic literature | Proposed shared pathways linking thyroid autoimmunity and CeD, including dysbiosis, increased intestinal permeability, immune activation, and micronutrient malabsorption. CeD served mainly as a comorbid intestinal autoimmune context rather than the primary evidence base. | Indirect, non-CeD-specific secondary evidence | Broad cross-disease narrative review; no systematic selection or formal quality appraisal; many probiotic and mechanistic data were animal-based; directionality between dysbiosis, permeability, and disease was uncertain; limited relevance to CeD-specific microbiota mechanisms. |

**Note:** Evidence directness was classified descriptively according to the study design and relevance to CeD. This table was intended to contextualize the biological interpretation of representative studies and was not designed as a formal risk-of-bias, GRADE, or certainty-of-evidence assessment.
